# Iridium and Ruthenium Complexes Bearing Perylene Ligands

**DOI:** 10.3390/molecules27227928

**Published:** 2022-11-16

**Authors:** Luca Mauri, Alessia Colombo, Claudia Dragonetti, Francesco Fagnani, Dominique Roberto

**Affiliations:** Dipartimento di Chimica, Università di Milano and INSTM UdR Milano, Via Golgi 19, I-20133 Milan, Italy

**Keywords:** iridium, ruthenium, perylene, absorption properties, emission properties, singlet oxygen, photodynamic therapy

## Abstract

The present review summarizes the work carried out mostly in the last decade on iridium and ruthenium complexes bearing various perylene ligands, of particular interest for bioimaging, photodynamic therapy, and solar energy conversion. In these complexes, the absorption spectra and the electrochemical properties are those of the perylene subunit plus those of the metal moiety. In contrast, the emissions are completely changed with respect to perylenes considered alone. Thus, fully organic perylenes are characterized by a strong fluorescence in the visible region, lifetimes of a few nanoseconds, and luminescence quantum yields approaching 100%, whereas perylene Ir and Ru complexes usually do not emit; however, in few cases, weak phosphorescent emissions, with lifetimes in the range of microseconds and relatively low quantum yields, are reported. This is due to a strong interaction between the perylene core and the heavy metal center, taking place after the excitation. Nevertheless, an important advantage deriving from the presence of the heavy metal center is represented by the ability to generate large amounts of singlet oxygen, which plays a key role in photodynamic therapy.

## 1. Introduction

**Figure 1:**
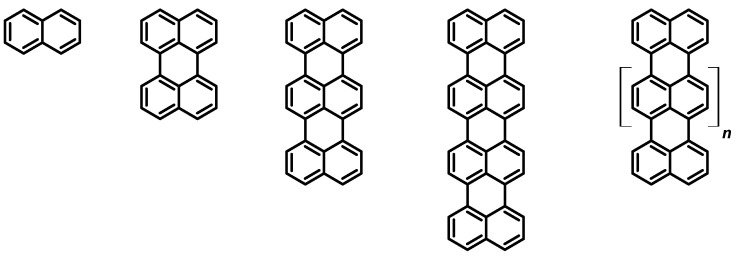
The following are shown from left to right: naphthalene, perylene, terrylene, quaterrylene, and a generical oligorylene.

Considering the wide variety of photoluminescent molecules, rylene dyes (see Figure 1) represent a family of organic molecules constituted by two or more naphthalene subunits connected in the peri-positions. Among the peculiarities of this family, we include high molar extinction coefficients [1] and large luminescence quantum yields, together with red-shifted spectra, as the number of naphthalene subunits in the rylene core is increased [2].

**Figure 2:**
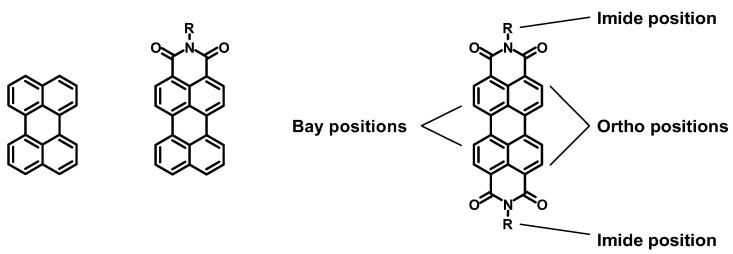
The following are shown from left to right: perylene, perylene monoimide (PMI), and perylene diimide (PDI) with the three functionalization positions.

Perylene is particularly interesting, thanks to its strong absorption and emission in the visible region and to its easy functionalization; thus, various substituents can be introduced to decorate the unsubstituted perylene core, or together with one or two imide groups in the peri-position (see Figure 2). In particular, perylene diimides (PDIs) have attracted the attention of many research groups because of their versatility in terms of functionalization and applications. In fact, several reviews describing the functionalization potential of this class of molecules are available in the recent scientific literature [3,4], together with many others describing the possible applications of PDIs in different fields, such as photocatalysis, solar energy conversion [5], medicine [6], sensors [7], and organic electronics [8]. Furthermore, the solubility and the spectroscopic properties of PDIs can be easily modulated by working on the three functionalization positions characterizing this polycyclic compound, namely two imide, four ortho (or shoulder), and four bay positions. The imide functionalization is not associated with the optical properties arising from the perylene core, but with the solubility of the derivative [9], whereas the ortho and bay functionalization are strongly related with the spectroscopic features of the PDI [10]. 

Although many studies about the synthesis and application of various organic perylene dyes have been reported in the literature, only a restricted number of papers describing coordination compounds containing a perylene core can be found [11,12,13]. Iridium and ruthenium complexes are particularly fascinating; they are widely employed in research fields, such as bioimaging, photodynamic therapy, and solar energy conversion. 

Bioimaging is a class of techniques which aims to visualize biological environments in real time in a non-invasive way. Of great interest is luminescent bioimaging, in which a luminophore is incubated into the biological environment, reaches one of its excited states through the proper excitation wavelength, and then images are taken through visualization techniques, such as confocal microscopy. 

Photodynamic therapy is also of growing interest. It is an anticancer treatment taking advantage of a luminophore which can be excited by an external source of radiation: once activated by light, the compound causes cytotoxic reactions leading to severe damages (or even the death) of the cells hit by the exciting radiation, without involving cells and tissues not exposed to the radiation. Considering the acting mechanisms, the excess energy of the excited state of the molecule is transferred to molecular oxygen, generating cytotoxic species through different processes; in this way, both reactive oxygen species (such as OH·, O_2_^−^, H_2_O_2_, etc.) or singlet oxygen (^1^O_2_) can be produced. Several examples of both organic and organometallic dyes based on iridium, ruthenium, and other metals bearing various ligands employed in these application fields have been widely described in the literature [14,15,16,17,18,19,20,21,22,23]. Photodynamic therapy represents an interesting and less invasive technique able to play a key role in the future of anticancer treatments. 

Moving to the field of solar energy conversion, dye-sensitized solar cells (DSSCs) represent an important attempt to produce efficient, cheaper, and greener photovoltaic devices. This kind of solar cell is not based on semiconductors, such as silicon, but instead exploits the spectral and electrochemical properties of a dye deposited over a photoanode made of a thin film of TiO_2_ over a conductive glass, such as fluorinated tin oxide (FTO); moreover, the cathode is generally made of fluorinated tin oxide on which a Pt counter electrode is deposited. Between the two electrodes, an electrolytic solution containing a redox couple (usually I^−^/I_3_^−^) is placed. The role of the dye is to absorb sunlight and to inject electrons into the titania layer, reaching an oxidized form. The electrons flow through an external circuit to the cathode and recombine with the oxidized redox mediator to create the reduced form, which reacts with the oxidized dye by reducing it, and restoring the molecules for another cycle. Several organic and organometallic dyes have been developed [24,25,26,27]. Indeed, Ru(II) complexes are particularly appealing, due to their high efficiencies (up to 10%). The most common example of a ruthenium dye employed as sensitizers in dye-sensitized solar cells is di-tetrabutylammonium cis-bis(isothiocyanato)bis(2,2′-bipyridyl-4,4′-dicarboxylato)ruthenium(II) (N719), which is used as a reference in this research field, but several other Ru-based dyes have also been widely described [28].

Up to now a review describing the characterization and the applications of iridium and ruthenium complexes bearing perylene subunits has never been published. Our work will provide a detailed summary of this research field.

## 2. Iridium Complexes Bearing Perylene Ligands

In this section, various studies, from 2009 to the present, describing the synthesis and characterization (both spectroscopic and electrochemical) of iridium complexes bearing various types of perylene ligands are reported. Although most of them were focused only on these types of characterization, in some cases tests in light-emitting electrochemical cells and in cells as sensitizers for photodynamic therapy were also made.

Luminescent iridium complexes have been studied for decades, and several iridium organometallic compounds bearing variously functionalized phenanthrolines, bipyridines, and other ligands were synthesized, characterized, and tested for different applications [29]. In this subsection, we report the papers, from 2009 to the present, discussing iridium complexes bearing perylene ligands.

**Figure 3:**
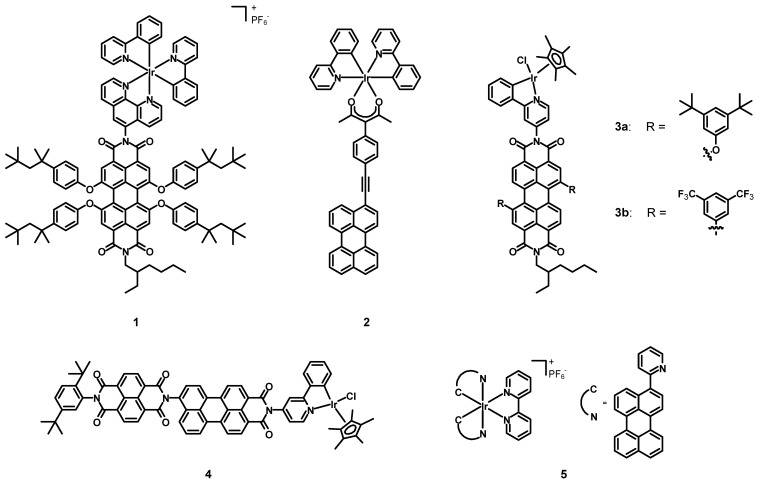
Structures of complexes **1**, **2**, **3a**–**b**, **4**, and **5**.

In 2009, Bolink, Lázaro, Santos et al. [30] synthesized and tested a novel perylene diimide-Ir(III) compound (**1**, structure in Figure 3 data in Table 1) in light-emitting electrochemical cells (LECs). This complex was constituted by an asymmetrical PDI bearing a phenanthroline (phen) ligand in the imidic position of the perylene, with two cyclometalating phenylpyridines (ppy) as additional ligands on the metal. Despite the deep-red emission and the high fluorescence quantum yield (Φ) of PDIs, their poor ability to act as hole conductors made them unsuitable for single-component LECs. On the other hand, the lack of deep-red emitting could be fulfilled by choosing a PDI-Ir(III) dyad, to take advantage of the synergy between the iridium moiety (which could act as a hole conductor) and the perylene core, being a red fluorescent emitter. The two subunits were covalently linked to improve the thermal stability and to avoid phase separation processes. Furthermore, the choice of bulky 4-*tert*-octylphenoxy groups was meant to minimize both the aggregation between the perylene cores and the interactions between Ir(III) centers.

Density functional theory (DFT) calculations suggested that the highest occupied molecular orbital (HOMO) and the lowest unoccupied molecular orbital (LUMO) stood on the PDI moiety with an energy gap of 2.41 eV, whereas the HOMO-1 and the LUMO+1 levels were on the Ir(III) subunit, with identical findings to those of the free complex (3.01 eV [31]). The two subunits were electronically decoupled because of the imide moiety and the angle between the PDI core and the phenanthroline, which hampered the conjugation. Compound **1** showed an emission band centered at 619 nm, similar to that of Lumogen^®^ F Red 305 (613 nm [32]); this emission in the red region differs from that of Ir(III) complexes (510–590 nm, yellow–green [31]). The high luminescence quantum yield (55 %) and short lifetimes (3.0 ns) observed when irradiating the PDI core indicated that compound **1** was suitable for applications in electroluminescent devices.

A LEC was assembled by first depositing a 100 nm active poly(3,4-ethylenedioxythiophene)-poly(styrenesulfonate) (PEDOT:PSS) layer, and then an 80 nm layer of **1** over an ITO (indium tin oxide) support through spin coating processes, followed by the deposition of an Al layer as a top contact. Small amounts of 1-butyl-3-methylimidazolium hexafluorophosphate (BMIMPF_6_) at a molar ratio 2:1 **1**: BMIMPF_6_ were added to decrease the turn-on time and driving voltage. The electroluminescence spectrum of **1** had the same shape of the photoluminescence one but appeared red-shifted of 25 nm because of a solid-state effect. An increase in the luminance and in the current density was also observed when a voltage of 3 V was applied. High current efficiency (2.5 cd A^−1^), maximum power efficiency (2.56 lm W^−1^), and external quantum efficiency (3.27%) were reached after 22 min of device operations, clearly indicating the possibility of employing compound **1** for LECs.

**Table 1:** Photophysical and redox properties of complexes **1**, **2**, **3a**–**b**, **4**, **5**, **6a**–**b**, **7**, and **8a**–**b**.

Complex	λ_abs_ (nm) (ε, M^−1^ cm^−1^)	λ_em_ (nm) (Φ (%); τ)	E_red_ (V)	E_ox_ (V)	Ref.
**1**		619 ^1^ (55; 3.0 ns)			[30]
**2**		475, 510 ^2^ (-; 2.6 ns ^3^)	−1.72 ^4,5^ (irr.)	+0.74 ^4,6^ +0.95 ^4,5^	[33]
**3a**	553 ^1^ 450 ^1^	576	−0.57 ^5,7^ −0.79 ^5,7^	+0.80 ^6,7^	[34]
**3b**	544 ^1^ 450 ^1^	579	−0.35 ^5,7^ −0.61 ^5,7^	+0.80 ^6,7^	
**4**	482 ^1^ 508 ^1^		−0.51 ^8,9^ −0.89 ^5,8^	+1.48 ^5,8^ +0.83 ^6,8^	[35]
**5**	269 (77,625) ^1^ 295 (38,905) ^1^ 308 (33,113) ^1^ 346 (13,804) ^1^ 458 (38,019) ^1^ 489 (42,658) ^1^ 522 (44,668) ^1^	570, 628 ^1^ 566, 623 ^10^			[36]
**6a**	470 (65,000) ^1^	745 ^1^ (<1; 33 μs) 744 ^11^ (-; 83 μs)	−0.82 ^5,14^ −1.22 ^5,14^	+1.46 ^14^ +1.42 ^14^	[37]
**6b**	470 (65,000) ^1^	780 ^1^ (6 ^12^, 11 ^13^; 4.2 μs) 763 ^11^ (-; 5.6 μs)	−0.85 ^5,14^ −1.23 ^5,14^	+1.02 ^6,14^	
**7**	241 (72,000) ^15^ 269 (55,000) ^15^ 322 (36,000) ^15^ 373 (32,000) ^15^ 443 (30,000) ^15^ 474 (40,000) ^15^ 560 (1641) ^15^	491, 516 ^15,16^ (52; 2.83 ns) 724, 827 ^15,17^ (1, -)	−0.58 ^14^ −1.22 ^14^ −1.75 ^14^	+0.86 ^14^ +1.40 ^14^	[38]
**8a**	359 ^1^ 375 ^1^ 394 ^1^ 466 ^1^ 497 ^1^ 582 ^1^	550 ^1^ (2, -)	−1.21 ^5^ −1.42	+0.54	[39]
**8b**	355 ^1^ 372 ^1^ 391 ^1^ 465 ^1^ 495 ^1^ 564 ^1^	615 ^1^ (28, -)	−1.14 ^5^ −1.33		

^1^ Measured in dichloromethane (DCM). ^2^ Measured in tetrahydrofuran (THF). ^3^ Measured in acetonitrile (ACN). ^4^ Using ferrocene/ferrocenium (Fc/Fc^+^) as an internal standard and converted to saturated calomel electrode (SCE) by the relation E_1/2_ (Fc/Fc^+^) = +0.38 V (ΔE_p_ = 60 mV) vs. SCE. ^5^ Process associated with the perylene subunit. ^6^ Process associated with the metal center. ^7^ Using Ag/AgCl as a reference electrode. ^8^ Using a SCE as an internal standard. ^9^ Process associated with the naphthalene diimide. ^10^ Measured in *n*PrCN at 77 K. ^11^ Measured at 77 K in 4:1 EtOH:MeOH. ^12^ Associated with the metal-to-ligand charge transfer (MLCT) transition. ^13^ Associated with the PDI ligand transition. ^14^ Using Fc/Fc^+^ as an internal standard. ^15^ Measured in CHCl_3_. ^16^ Measured in dilute solution (1 × 10^−5^ M). ^17^ Measured in concentrated solution (1 × 10^−2^ M).

In 2011, Castellano, Ziessel et al. [33] synthesized the Ir(III) complex **2** (structure in Figure 3, data in Table 1) and determined its spectroscopic properties. This compound was characterized by two simple cyclometalating ppy ligands and a chelating acetylacetonate (acac) functionalized with a conjugated perylene subunit. The Ir(III) center was introduced in order to act as a control molecule, while the acac–perylene ligand was present to localize the excited triple state on the extended π-conjugated hydrocarbon core.

The electrochemical activity of compound **2** showed a reversible one-electron and metal-based oxidation at +0.74 V vs. ferrocene/ferrocenium (Fc/Fc^+^) and converted to saturated calomel electrode (SCE). Two additional oxidation processes at +0.95 V (reversible) and +1.22 V (irreversible), and two reductions at −1.93 and −1.72 V (both irreversible) were observed and assigned to the perylene core. The absorption spectrum showed a broad absorption band in the region between 400 and 510 nm, with molar extinction coefficients up to 20,000 M^−1^ cm^−1^, in addition to a strong absorption band associated with the intraligand (^1^π-π*) transition between ppy ligands below 300 nm. Concerning the emission, the spin-allowed metal-to-ligand charge-transfer (^1^MLCT) and the ^1^π-π* transition strongly overlap; therefore, it was impossible to selectively excite only one of the two components of the molecule. The main emission bands were those of the perylene group at 475 and 510 nm, and the lifetime of 2.6 ns was about 40% shorter than that of a perylene core in a completely organic system. The spin-forbidden metal-to-ligand charge-transfer (^3^MLCT) luminescence was quantitatively quenched giving way to that of the perylene subunit. Transient absorption measurements on oxygen-free solutions were carried out to obtain information about the processes taking place upon photoexcitation. When complex **2** was excited with 420 nm laser pulses, the main emission peaks corresponded to the singlet state, rapidly turning into the corresponding excited triplet perylene. Weaker triplet–triplet energy transfer processes with a longer lifetime were hidden by the more intense perylene transitions. The comparison of the energetics with the molecular orbitals showed that the population of the ^3^MLCT state was followed by a triplet–triplet energy transfer to the lower-lying triplet state, which decayed to the ground state. Compound **2** represent a promising candidate for solar fuel photochemistry, singlet oxygen production, and photon up-conversion based on triplet–triplet annihilation.

In 2012, Wasielewski et al. [34] published the new PDI-Ir(II) complexes **3a**–**b** (see Figure 3, data in Table 1) The iridium cation was coordinated to a pentamethylcyclopentadienide and a chloride, together with a cyclometalating ppy functionalized with a perylene diimide. Two bay positions were functionalized with electron-donating 3,5-di-*tert*-butylphenoxy groups (**3a**) or with electron-withdrawing 3,5-di(trifluoromethyl)phenyls (**3b**).

The main absorption bands were placed at 553 and 544 for **3a** and **3b** respectively, indicating a poor interaction between the perylene core and the Ir(III) moiety. The electrochemical characterization highlighted two reversible reductions of the PDI chromophore and an irreversible Ir(III)-Ir(IV) oxidation. Time-resolved spectroscopy experiments on **3a** in dichloromethane (DCM) and in toluene using 150 fs-550 nm pulses demonstrated the formation of a PDI radical anion, which was confirmed by the appearance of an absorption peak at 725 nm, and a charge separation evidenced by the disappearance of the stimulated emission at 620 nm; furthermore, **3b** gave a PDI radical anion, confirmed by a new absorption peak at 745 nm. Rapid charge recombination processes were suggested in both cases by the biexponential decay of the PDI radical anion, while transient absorption spectra demonstrated the presence of triplet excited PDIs in toluene. Compounds **3a**–**b** showed interesting photophysical properties and represent promising candidates for the catalytic oxidation of water.

In 2012, Wasielewski et al. [35] synthesized and characterized the new PDI-Ir(III) complex **4** (structure in Figure 3, data in Table 1). The structure of the Ir(III) subunit was exactly the same as in complexes **3a**–**b**, but the ppy ligand, instead of having a bay functionalized PDI, was decorated with a perylene monoimide (PMI), which presented a naphthalene diimide (NDI).

The UV–Vis spectrum in DCM was the superimposition of those of NDI (between 350 and 400 nm) and PMI (482 and 508 nm). The perylene fluorescence was quenched after metalation, suggesting an electron transfer involving this subunit and the Ir(III) center. Electrochemical experiments showed two oxidations at +1.48 V vs. a saturated calomel electrode (SCE) (PMI) and +0.83 V (Ir(III)/Ir(IV)), and two reductions at −0.51 V (NDI) and −0.89 V (PMI). From time-resolved spectroscopy in DCM with 150 fs-500 nm pulses, a broad excited state absorption emerged, ranging from 525 to 700 nm with a sharp peak at 610 nm. The subsequent decay to two bands at 540 and 610 nm indicated the charge separation yielding in the NDI radical anion-PMI-Ir(IV) species, which decayed in 17.2 ± 0.1 ns. Compound **4** is interesting from the photophysical point of view and can become a promising candidate for applications in photocatalysis.

In 2014, Sun et al. [36] synthesized and characterized the novel Ir(III) complex **5** (see Figure 3, data in Table 1) with a simple bipyridine (bpy) and two cyclometalating perylene ligands aimed at extending the π-conjugation.

The absorption spectrum of **5** in DCM showed broader and red-shifted bands with respect to the corresponding ligand, due to the delocalization of ligand-centered π-orbitals mediated by the Ir(III) *d* orbitals, with the increased planarity of the C^N ligand after the coordination to the metal. The strong absorption bands between 250 and 550 nm were associated to spin-allowed ^1^π-π* transitions, while the tail above 550 nm was attributed to spin-forbidden metal-to-ligand and ligand-to-ligand transitions. The major absorption bands around 500 nm were due to the extended π-conjugation, while the transition at 308 nm was associated to ^1^π-π* and spin-allowed ligand-to-ligand charge transfer transitions (^1^LLCT), with a contribution from the ^1^MLCT transition. The presence of the metal center dramatically red-shifted the emission of complex **5** (570 and 628 nm) with respect to the perylene ligand (470 and 496 nm). The emission intensity of complex **5** was very weak because of the strong rate constant of the nonradiative decay of the spin-forbidden ligand-to-ligand charge transfer transition (^3^LLCT); for this reason, the emission lifetime could not be determined. The emission spectra in butyronitrile (*n*PrCN) at 77 K showed a blue shift of few nm because of the rigidochromic effect of the solid matrix. Transient absorption studies in acetonitrile highlighted the involvement of charge-transfer processes.

**Figure 4:**
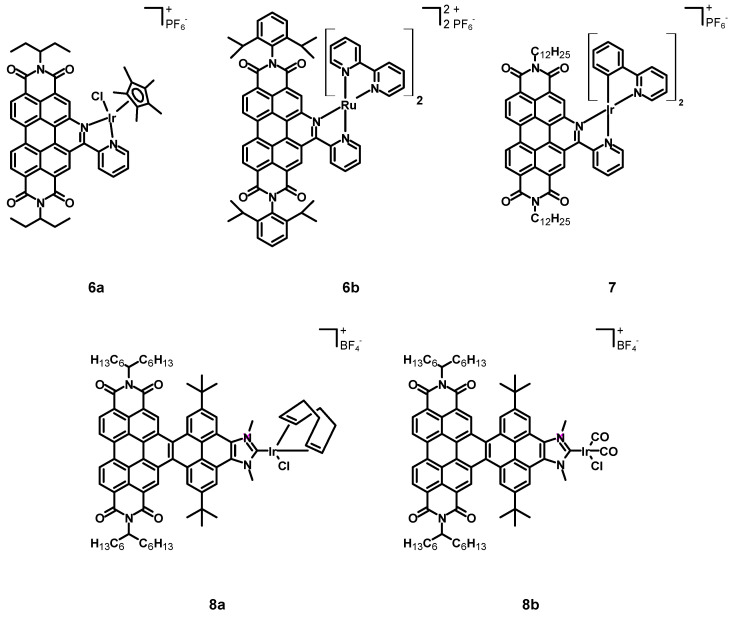
Structures of complexes **6a**–**b**, **7**, and **8a**–**b**.

In 2015, Steffen, Würthner et al. [37] published a new perylene diimide complex of iridium and a related ruthenium complex (**6a**–**b**, see Figure 4, data in Table 1). In this case, the perylene core and the iridium center were not two linked independent subunits, but a perylene-based chelating ligand was synthesized exploiting functionalization of bay positions. The other ligands were chloride and pentamethylcyclopentadienide.

Cyclic voltammetry studies showed two reversible PDI reductions at −0.82 V and −1.22 V vs. Fc/Fc^+^, less negative than those of the PDI ligand alone, clearly indicating an easier reduction after coordination to the metal centre, however accompanied by a more difficult oxidation of the latter at +1.46 V, associated with the strong electron-withdrawing character of the electron-poor PDI core. The UV–Vis spectrum of complex **6a** was characterized by a strong absorption band around 470 nm, and several bands were observed between 250 and 420 nm, due to MLCT transitions; in this spectral window, the absorptivity was almost constant and had a molar extinction coefficient of about 30,000 M^−1^ cm^−1^. The HOMO–LUMO gap of **6a** was 2.34 eV, the S_1_ state was a pure intra-ligand charge-transfer (ILCT) state, while the transitions between 370 and 420 nm were MLCT. The emission spectrum showed the quenching of the PDI emission (observed at 484 nm for the ligand alone), together with a long-living emission centred at 745 nm originating from a ^3^IL transition. The emission lifetime of **6a**, measured in degassed DCM, was 33 μs, shorter than the phosphorescence lifetime of other PDIs because of the stronger spin-orbit coupling. The phosphorescence quantum yield was lower than 1%, this being due to the heavy iridium atom, which favoured the spin-forbidden S_1_-T*_n_* and T_1_-S_0_ transitions.

In 2017, Chao, Gasser et al. [40] tested complexes **6a** and **6b** as photosensitizers for photodynamic therapy (PDT) because of their interesting spectroscopic properties, long emission lifetimes, and ability to absorb light up to 600 nm, close to the PDT window (600–1000 nm) that represents a range of less harmful and more penetrating wavelengths.

Firstly, the chemical stability was assessed through ^1^H-NMR studies, and it emerged that compound **6a** was subject to the substitution of the chloride anion by a water molecule. However, despite aquation, **6a** was stable enough for applications in PDT. The photostability was studied by irradiating an acetonitrile solution of **6a** at 420 nm for 15 min, confirming the possibility to employ these compounds for PDT. Then, the ability to generate singlet oxygen (^1^O_2_) was determined by measuring its phosphorescence quantum yield at 1274 nm both upon irradiation (at 420 nm) of an acetonitrile solution of **6a** (direct method) and in the presence of an imidazole derivative to trap singlet oxygen, forming an adduct that quenched the luminescence of a probe molecule (indirect method). The two methods confirmed that **6a** was able to generate ^1^O_2_ with high quantum yields ranging from 85 to 87%. The quantum yield was much lower (21%) when working with a phosphate buffer saline (PBS) solution of **6a**, as a consequence of its much lower solubility in this medium. The potential as photosensitizers for PDT was determined through biological tests on different cell lines (A2780, A2780R, HeLa, MRC-5). The dark toxicity was determined through the incubation of a 4 μM solution of **6a** with PS 5-aminolevulinic acid (5-ALA), while the phototoxicity was tested after 4 h of incubation and after 20 min of irradiation with 420 nm light. While the ligand alone did not show any phototoxicity, complex **6a** showed quite low IC_50_ values, ranging from 170 ± 50 nM to 520 ± 100 nM, resulting in phototoxic indexes (i.e., the ratio between the IC_50_ measured after 4 h without irradiation and that after the irradiation for 10 min) from 7.8 to 23. The dark toxicity was much lower than the phototoxicity, with IC_50_ values being between 2.8 ± 0.9 and more than 4 μM. The position of **6a** in the cell was determined through confocal microscopy, and a weak luminescence demonstrated its homogeneous distribution in the cytoplasm. Furthermore, ICP-MS analysis confirmed the presence of 0.81 nmol of Ir per mg of protein, mostly located in the mitochondria (0.64 nmol per mg of protein). Thanks to its stability, the ability to produce singlet oxygen and the high phototoxicity, complex **6a** represents a promising candidate for PDT.

In 2018, Wang et al. [38] synthesized the cyclometalated iridium complex **7** (structure in Figure 4, data in Table 1) bearing two ppy and a perylene ligand analogous to that employed for compounds **6a**–**b**, except for the imidic substituent that was a dodecyl chain.

Electrochemical measurements showed oxidation potentials between +0.80 and 1.40 V vs. Fc/Fc^+^, reduction potentials in the range between −0.50 and −1.80 V, and a HOMO–LUMO gap of 1.44 eV. The DFT and TDDFT calculations displayed that the HOMO was delocalized on the cyclometalating ligands while the LUMO was on the PDI ligand, with a HOMO–LUMO gap of 1.90 eV, and suggested a charge-transfer transition, associated with the lowest absorption band at 653 nm, from the cyclometalating ligands to the ancillary PDI ligand. The main absorption bands at 320 and 480 nm suggested the predominance of LC transitions mostly of the PDI core. The absorption spectrum showed intense absorption bands between 200 and 500 nm, associated with π-π* transitions of the PDI and the cyclometalating ligand. Moreover, a much weaker absorption band at 560 nm was attributed to MLCT and ILCT transitions. The emission spectrum of a 10^−5^ M solution of compound **7** in chloroform showed two emission maxima at 491 and 516 nm, with an emission lifetime of 2.83 ns, slightly more red-shifted than those of the PDI ligand; a weak emission band in the near-infrared at 747 nm was observed and associated to the MLCT transition. A strong red-shift to 827 nm was observed when the concentration was raised to 10^−2^ M. Broader and blue-shifted emission bands were observed at 77 K because of the rigidochromic effect taking place at that temperature. The fluorescence quantum yield from LC transitions (52%) was measured in a diluted 10^−5^ M solution of **7**, whereas the phosphorescence quantum yield (0.01%) was observed in a more concentrated solution (10^−2^ M). The novel cyclometalated complex **7** shows an interesting dual emission because both fluorescence and phosphorescence are detected; it represents a starting point to develop new phosphorescent materials bearing perylene cores.

In 2022, Alonso, Peris, Poyatos et al. [39] published two novel N-heterocyclic carbene (NHC) complexes **8a**–**b** (structure in Figure 4, data in Table 1). The NHC ligand consisted of a 1,3-dimethylimidazolium tetrafluoroborate condensed with a core-extended PDI through a pyrene subunit, in order to generate a nanographene ligand. The other ligands were a chloride in both **8a** and **8b**, a cyclooctadiene in compound **8a**, and two carbon monoxide molecules in **8b**.

Cyclic voltammetry studies showed the redox properties of **8a**–**b**, as two different reversible redox processes were observed in both cases, the first around −1.20 V vs. Fc/Fc^+^ associated with the formation of a neutral radical species and with the reduction in the PDI moiety, and the second around −1.40 V associated with a doubly reduced negative species. The carbonylated complex **8b** showed less of a negative reduction potential than **8a** because of the coupling between the metal center and the perylene core. An irreversible oxidation was only observed in case of **8a** at +0.54 V. The HOMO–LUMO gaps of **8a**–**b** were 2.13 and 2.20 eV, respectively. The UV–Vis spectrum of **8a** showed two main absorption maxima at 360 and 497 nm. On the other hand, spectroelectrochemical experiments in DCM of the same complex showed two other maxima at 417 and 682 nm, assigned to the one-electron reduced complex and indicating that these processes are associated with the perylene core. The further reduction provoked the disappearance of the signal at 682 nm and the appearance of a new band at 631 nm. Conversely, infrared spectroelectrochemical experiments were carried out to understand if the reduction of the PDI ligand could have an effect on the C–O stretching frequencies in the case of compound **8b**. The reduction in the PDI provoked a remarkable decrease in the intensities of the C–O stretching bands at 2071 and 1988 cm^−1^, and the appearance of two new bands at 2049 and 2053 cm^−1^, indicating that this NHC ligand has a strong electron-donating character. Complexes **8a**–**b** showed emission maxima at 550 and 615 nm, respectively, and, similar to other perylene-Ir complexes, emission quantum yields (2 and 28%) were lower than the free ligand (54%). Compounds **8a**–**b** show interesting spectroscopic and electrochemical processes and represent promising candidates for applications in catalysis and other research fields, such as supramolecular chemistry.

## 3. Ruthenium Complexes Bearing Perylene Ligands

A wide variety of ruthenium complexes bearing several ligands have been studied for decades [29], not only from a spectroscopic point of view but also for their application in solar energy conversion devices, sensors for the detection of pollutants, and PDT. Here we report the studies from 2005 to present, describing ruthenium complexes bearing perylene ligands.

**Figure 5:**
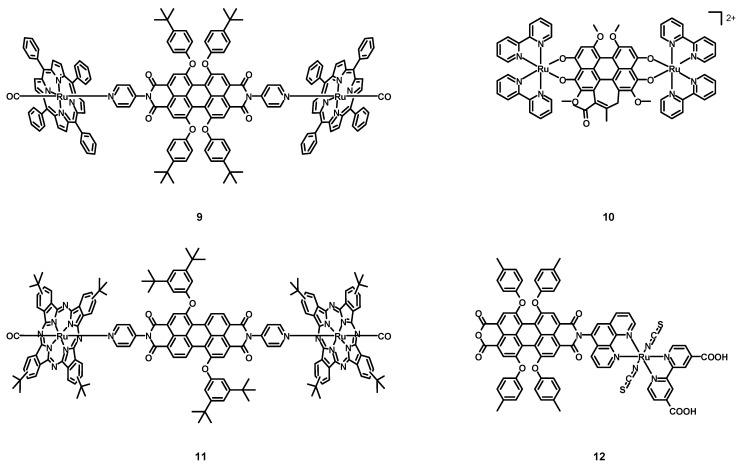
Structures of complexes **9**, **10**, **11**, and **12**.

In 2005, Iengo, Scandola, Würthner et al. [41] synthesized the new perylene–porphyrin complex **9** (structure in Figure 5, data in Table 2), taking advantage of the stability of side-to-face porphyrin assemblies and of the interesting spectroscopic and electrochemical properties of PDIs. The PDI was characterized by two monodentate pyridine ligands in the imidic position and by four 4-*tert*-butylphenoxy groups in the bay positions. The employed porphyrin was functionalized with four phenyl rings, and the remaining ligand on the metal center was a CO molecule.

The absorption spectrum of **9** in DCM corresponded to that of the PDI plus the porphyrin–Ru complex considered alone. Even the electrochemical properties were estimated to be similar to those of the two independent subunits, namely two reversible reduction steps at −0.62 and −0.77 V vs. SCE and an irreversible oxidation at +1.6 V for the PDI, and two reversible oxidation steps at +0.81 and +1.36 V for the Ru–porphyrin. The excitation wavelength of 585 nm was absorbed practically only by the perylene core but, because of the strong heavy atom effect, the fast intercomponent electron transfer yielding the PBI^−^/Ru^+^ charge-separated state, and the intercomponent energy transfer to the isoenergetic Ru-porphyrin S_1_ state, the high fluorescence observed for the ligand alone (λ_max_ = 618 nm, Φ = 96%) was quenched almost completely. When **9** was excited with 530 nm light, the absorption was mostly due to the porphyrin core (62%), with only a weak fluorescence at 620 nm being observed, without the phosphorescence at 726 nm associated to the porphyrin. Ultrafast time-resolved experiments with a 585 nm excitation wavelength demonstrated that in the short timescale (τ < 50 ps), the spectral changes were those of the absorptions of the PDI core, of the PDI radical anion at 780 nm and of the Ru–porphyrin radical cation at 630 nm, indicating the charge separation. On the other hand, on a longer timescale (up to 1 ns) the bands associated with the charge separation disappeared, due to a charge recombination resulting in the restoration of the ground state. Working with an excitation wavelength of 530 nm, also a 460 nm absorption band was observed in the early timescale (τ < 68 ps) related to the T_1_ excited state reached by the Ru–porphyrin. On a longer timescale, an energy transfer process from the Ru–porphyrin to the PDI, yielding PDI in the T_1_ excited state followed by the decay to the ground state, was observed. Complex **8** showed interesting photophysical properties, especially the ability to generate a triplet excited PDI that cannot be observed in metal-free perylene diimides because of the high fluorescence quantum yields.

In 2005, Wang, Zhang, Zhou et al. [42] synthesized and characterized the hypocrellin B-based Ru(II) complex **10** (see Figure 5, data in Table 2), in order to obtain a more efficient and water-soluble sensitizer for PDT. Hypocrellin B, i.e., a perylene derivative with an extended core, was coordinated to two ruthenium atoms, together with four bpy chelating ligands.

Differently from the apolar hypocrellin B, complex **10** was soluble in polar solvents, such as water, ethanol (EtOH), and dimethyl sulfoxide (DMSO). The UV–Vis spectrum of **10** in EtOH was characterized by a strong absorption band centered at 647 nm, so in the PDT window. The ability to form singlet oxygen was tested in an oxygen-saturated DMSO solution in presence of 2,2,6,6-tetramethyl-4-piperidone (TEMP) because of its ability to form a 4-oxo-2,2,6,6-tetramethyl-1-piperidinyloxy free radical (4-oxo-TEMPO) in the presence of ^1^O_2_, whose presence was detected through ESR spectroscopy. Compound **10** represents an interesting candidate for PDT for its solubility in organic solvents, its ability to generate singlet oxygen, and the absorption maximum in the PDT window.

In 2006, Guldi, Torres et al. [43] synthesized and characterized the perylene-phthalocyanine–ruthenium complex **11** (structure Figure 5, data in Table 2). Differently from compound **9**, only two bay positions were functionalized with bulky 3,5-di-*tert*-butylphenoxy groups.

Also in this case, the absorption spectrum of the compound was that of the PDI ligand plus that of the phthalocyanine–Ru complex and was characterized by two main absorption bands at 296 and 652 nm, associated with the phthalocyanine absorption and with weaker bands around 500 nm related to the absorption of PDI. Electrochemical experiments showed two reversible oxidations at +0.66 and +1.42 V for the phthalocyanine complex alone, and one reversible reduction taking place at −0.6 V for the PDI alone. The electrochemical behaviour of **11** mimicked that of the two distinct components, but with an oxidation peak shifted to +0.74 V. As with compound **9**, complex **11** showed a very weak fluorescence, independently on the excitation of the PDI or the phthalocyanine cores at 560 or 655 nm, with quantum yields equal to 0.1% and 0.011%. On the other hand, the PDI and the phthalocyanine–Ru complex alone were characterized by emission bands at 587 and 660 nm with 75% and 0.015% quantum yields, respectively. Transient absorption spectroscopy showed a bleach centered at 560 nm and a singlet lifetime of 6.0 ± 0.5 ns for the PDI, while a maximum at 555 nm and two minima at 585 and 650 nm, respectively, indicated an efficient intersystem crossing observed for the phthalocyanine–Ru subunit. The presence of a heavy atom improved the efficiency of the intersystem crossing and a short singlet lifetime of 60 ± 0.2 ps was determined, with a triplet lifetime of 12 μs in the absence of oxygen. For what concerns complex **11**, in the short timescale the trend was similar to those of the two isolated subunits, with really fast singlet deactivations of 19 and 45 ps, and efficient intersystem crossing processes with constants of 1.6 × 10^−8^ and 1.6 × 10^−10^ s^−1^ for the PDI and the phthalocyanine, respectively. Another interesting donor-acceptor Ru–perylene complex was synthesized and fully characterized and, thanks to the two ruthenium atoms, it showed interesting photophysics that could not be observed in the absence of them.

In 2008, Tian, Zhu et al. [44] published the perylene–ruthenium complex **12** (structure in Figure 5, data in Table 2) and tested it in dye-sensitized solar cells (DSSCs). This compound was characterized by an anchoring 2,2′-bipyridine-4,4′-dicarboxylic acid ligand, together with two monodentate thiocyanates and a phenanthroline–perylene ligand. The latter was an asymmetrical perylene derivative with only one functionalized imidic position with a chelating phenanthroline and four 4-methylphenoxy groups in the bay positions. Differently from the commonly used dyes in DSSCs, that consist of an electron-rich donating group of atoms able to harvest a lot of sunlight and an electron-poor acceptor and anchoring group connected via a π-conjugated bridge, compound **12** was a simple Ru(II) dye functionalized with a non-conjugated electron-poor PDI core.

Compound **12** exhibited three absorption bands with high molar extinction coefficients, attributed to the absorption of the perylene. The HOMO and LUMO levels were 0.92 V vs. NHE and −0.75 V, respectively, more positive than the iodine redox potential (≈0.4 V) and more negative than the conduction band of the TiO_2_ semiconductor; as a consequence, **12** was able to inject electrons in the TiO_2_ layer and to be reduced by classical I^−^/I_3_^−^ redox electrolytes.

A DSSC was assembled by employing a TiO_2_ semiconductor over a fluorine-doped tin oxide (FTO) conductive glass and a platinum counter electrode. The chosen redox electrolyte was a solution of LiI (0.3 M) and I_2_ (0.03 M) in 9:1 *v*/*v* acetonitrile:3-methyl-2-oxazolidinone. The cell was tested under 200 W m^−2^ irradiation, showing an open circuit potential (V_OC_) of 0.42 V, a short-circuit current (J_SC_) of 1.35 mA cm^−2^, and a fill factor (FF) of 0.62, hence, resulting in an efficiency of 1.75%. Despite the strong absorption bands, the high electron-withdrawing character of the imide and the anhydride groups provoked an unbeneficial electron flow towards the perylene subunit which competed with the beneficial flow towards the anchoring ligand, hence, to the TiO_2_ layer, resulting in a poorer electron injection, thus, in a low J_SC_ and in a poor efficiency; the adsorption on the TiO_2_ was poor (5.12 × 10^−8^ mol cm^−2^) because of the steric hindrance of the perylene subunit. Although complex **12** was not a successful attempt to synthesize a performing sensitizer for DSSCs because of the strong electron-withdrawing character of its functional groups, the absence of conjugation between the perylene core, and the ancillary ligand and its hindrance, the right optimization of this compound could lead to better performing dyes [45].

**Figure 6:**
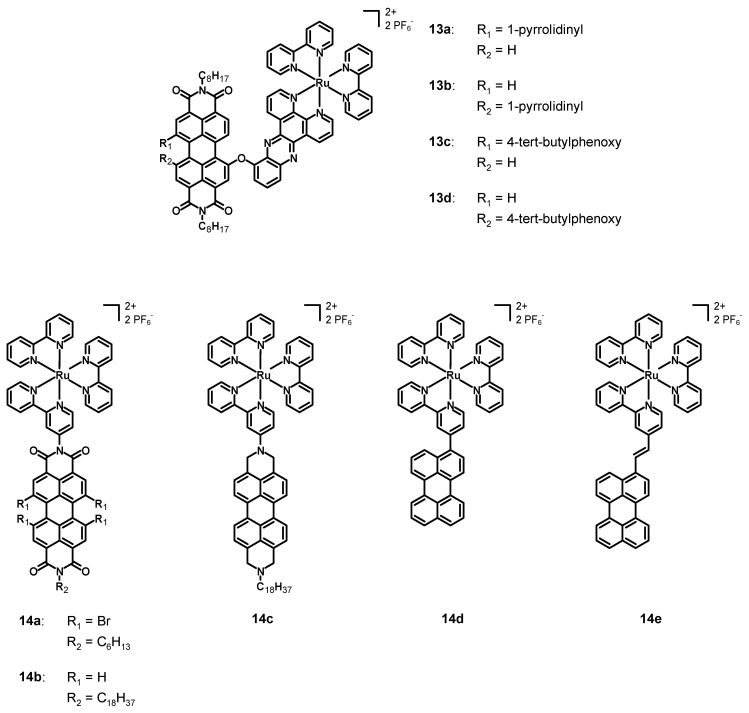
Structures of complexes **13a**–**d** and **14a**–**e**.

In 2013, Dubey et al. [45] synthesized and characterized the four perylene diimide–polypyridine Ru(II) complexes **13a**–**d** (see Figure 6, data in Table 2). The perylene diimide was characterized by simple *n*-octyl chains in the imidic positions and by an asymmetrical bay functionalization. Compounds **13a**–**b** had a 1-pyrrolidinyl substituent, while **13c**–**d** a 4-*tert*-butylphenoxy group; each complex presented a chelating dipyrido [3,2-a:2′,3′-c]phenazine (dppz) connected through an ether bond to the perylene core, while the other ligands were simple bpy. The main difference between **13a** and **13b**, and between **13c** and **13d**, was the different bay functionalization, as follows: 1,7 in the case of **13a** and **13c**, but 1,6 in the case of **13b** and **13d**. Compounds **13a**–**b** were characterized separately, because the separation of the 1,6 from the 1,7 isomer was feasible and was carried through column chromatography after the synthesis of the perylene ligand. On the other hand, this was not possible for the phenoxy-substituted PDIs and, hence, a mixture of complexes **13c**–**d** was characterized. The pyrrolidinyl-substituted PDIs were chosen in order to take advantage of the broad and strong absorption in the near IR, while the phenoxy-substituted ones were chosen due to their high solubility, and with the aim of observing the direction of energy transfer processes in the presence of different bay substituents.

The absorption spectra of **13a**–**d** in acetonitrile were characterized by a strong MLCT band centered at 438 nm associated with the ruthenium complex, and by a broader and stronger absorption band between 550 and 750 nm (related to the PDI) for complexes **13a**–**b** and between 450 and 590 nm (centered at 545 nm) for **13c**–**d**. The emission spectra of **13a** and **13b** were constituted by a broad emission band ranging from 700 to 850 nm and with fluorescence quantum yields of 0.33 and 0.39%. Compounds **13c**–**d** were characterized by an emission band ranging from 550 to 750 nm (centered at 580 nm) and by a fluorescence quantum yield of 2%. From the photophysical characterization it emerged that the singlet excited state was quenched, and transient absorption studies demonstrated that, after the excitation of the Ru complex subunit, the PDI reached the triplet state through a charge-transfer state with a higher energy than that of the triplet state, whose direction was dependent on the substituents in bay positions. In fact, it was directed from the Ru center to the PDI in case of complexes **13c**–**d** and, vice versa, from the PDI to the Ru subunit in case of complexes **13a**–**b**. On the other hand, when the PDI was excited, the charge-transfer was observed only for complexes **13c**–**d**. The electrochemical characterization showed that the phenoxy-substituted **13c**–**d** had three reductions at −0.55 V, −0.80 and −0.97 V vs. AgCl, and two oxidations at +1.42 and 1.70 V. Higher values were observed for **13a** and **13b**, which were characterized by first reduction potentials of −0.67 V, second reduction potentials of −0.87 V, and third reduction potentials of −0.99 and −1.00 V, respectively. The oxidation potentials were +0.90 and +0.94 V (first oxidation potential), and +1.38 and +1.40 V (second oxidation potential). Complexes **13a**–**d** showed interesting photophysics, especially thanks to the marked directionality of the charge-transfer processes caused by the different bay functionalization. Moreover, the phenoxy substituent had an interesting ability to accept electrons, while the pyrrolidinyl group was a good electron donor, confirming again the directionality of the charge-transfer process. The presence of a heavy Ru center close to the PDI core was also of crucial importance for observing the formation of a triplet excited PDI and a very efficient intersystem crossing.

In 2013, Hirose et al. [46] published five new bpy–ruthenium complexes (**14a**–**e**, structure in Figure 6, data in Table 2), bearing two unsubstituted 2,2′-bipyridines and a 2,2′-bipyridine functionalized with a perylene. In particular, the perylenes in complexes **14a**–**b** were diimides with different bay functionalization, while they were a simple core in compounds **14d**–**e**. On the other hand, **14c** was analogous to **14b**, but the imidic carbonyl groups were reduced to the corresponding methylenes.

The UV–Vis spectra of **14a**–**b** in *N,N*-dimethylformamide (DMF) were characterized by three absorption maxima at about 450, 500, and 530 nm with high molar extinction coefficients associated with π-π* transitions characteristic of the PDI, with a contribution of MLCT transitions at around 420 nm. The functionalization with four bromine atoms provoked a slight red-shift of the absorption of **14a** with respect to **14b**. The absorption of **14c** was much simpler and characterized by three bands at 431, 457 and 520 nm, slightly blue-shifted with respect to **14a**–**b**. The emission spectra of **14a**–**b** and **14e** in acetonitrile were characterized by an emission maximum at 569, 567, and 538 nm, respectively. Those of compounds **14c**–**d** consisted of two emission bands at 583 and 637 nm for **14c** and at 469 and 495 nm for **14d**. The latter had an emission similar to that of the free ligand, hence, the MLCT emission was quenched by the triplet excited state of the ligand. The novel synthesized perylene-Ru(II) complexes showed interesting photophysical properties and are promising candidates for applications in DSSCs and photocatalysis.

**Figure 7:**
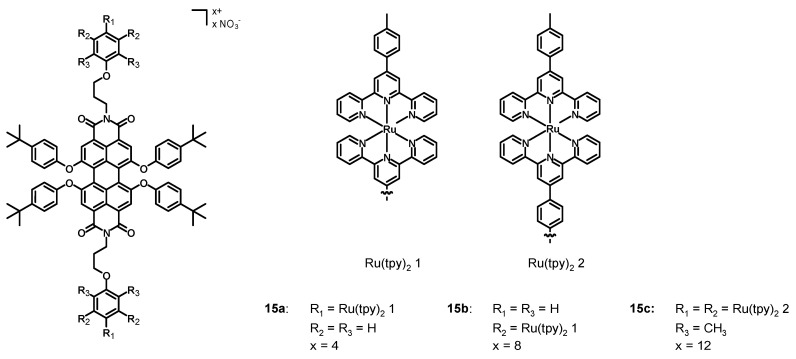
Structure of complexes **15a**–**c**.

In 2013, Newkome, Wesdemiotis et al. [47] synthesized the perylene diimide Ru(II) complexes **15a**–**c** (Figure 7, data in Table 2), in which the bay positions were functionalized with 4-*tert*-butylphenoxy groups and the imidic functionalizations were decorated with two, four, and six ruthenium terpyridine (tpy) complexes.

The UV–Vis spectra in DMF were constituted by strong ^1^MLCT absorption bands at 497 nm, ^1^LC at 288 and 313 nm, and one at 570 nm associated with the perylene π-π* transition. The increasing molar extinction coefficient of the bands at 288, 313, and 497 nm passing from **15a** to **15c** was a consequence of the increasing number of metal centers. Compounds **15a**–**c** represent a successful attempt to synthesize PDI-Ru(tpy)_2_ derivatives with broad and intense absorption bands. This characteristic, together with the wide functionalization possibilities, could lead to promising candidates for DSSCs.

**Figure 8:**
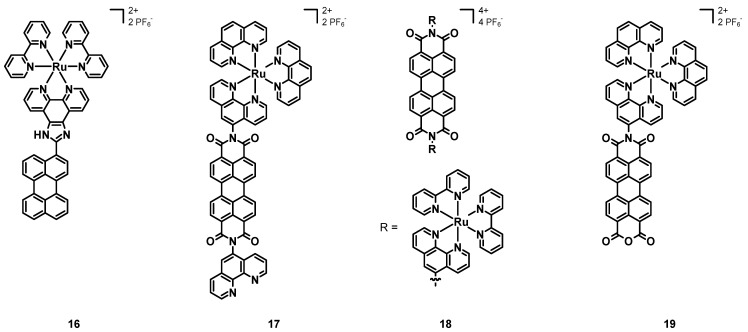
Structures of complexes **16**, **17**, **18**, and **19**.

In 2013, Wang et al. [48] synthesized and characterized the Ru–perylene complex **16** (structure in Figure 8, data in Table 2), having two simple bpy ligands and a 1*H*-imidazo[4,5-f]-1,10-phenanthroline functionalized with a simple perylene subunit.

The absorption spectrum of **16** was characterized by a strong band at 438 nm attributed to a MLCT transition, and by two intense bands around 300 nm associated with IL π-π* transitions. The emission was constituted by a single band at 610 nm with a remarkable red-shift due to the extended π-conjugation of the perylene core. The luminescence quantum yield was 32%, with a lifetime of 2.82 μs. Electrochemical studies showed a reversible oxidation at +1.05 V and an irreversible reduction at −1.22 V.

The previously cited work of Steffen, Würthner et al. [37] also described the Ru–perylene complex **6b** (Figure 4, data in Table 1), characterized by a perylene ligand obtained through an extension of the perylene core and by two simple bpy.

Electrochemical studies showed two redox potentials of −1.06 and −1.34 V vs. Fc/Fc^+^ for the PDI ligand alone and of −0.85 V and −1.23 V for **6b** and, hence, an easier reduction in the presence of a metal center and a more difficult oxidation at +1.02 V related to the electron-withdrawing character of the PDI core. Compound **6b** showed a broad absorption band at 520 nm associated with a MLCT transition and a strong peak around 300 nm due to the bpy ligands. Other bands at about 470 nm were those of the PDI ligand. The DFT calculations resulted in a HOMO–LUMO gap of 2.39 eV, and the partial MLCT character of the transition at 520 nm, which was mainly an ILCT. The emission spectrum of **6b** showed the almost complete quenching of luminescence in the presence of oxygen and an emission in the near IR between 750 and 1000 nm in the absence of it, with a lifetime of 4.2 μs. A remarkable phosphorescence quantum yield of 11% was observed for **6b**. Although Ru is a lighter element than Ir, the spin-orbit coupling was more pronounced in **6b** than in **6a**, this being attributed to the higher degree of MLCT transitions in the first complex.

In 2016, Carlos, Martinho et al. [49] published the novel perylene–Ru(II) complex **17** (structure in Figure 8, data in Table 3), constituted by a simple PDI scaffold with two phenanthrolines in the imidic positions; only one of them was coordinated to the ruthenium cation, bearing as the other ligands two unsubstituted phenanthrolines.

The UV–Vis characterization showed three intense bands associated with the absorption of the PDI between 450 and 550 nm, and a broad band centered at about 450 nm related to the ruthenium complex. Both the emission spectra obtained with λ_ex_ = 413 and 450 nm showed an intense emission centered at 600 nm, but only the second one had a shoulder peak at 525 nm, associated with the excitation of the Ru–phen complex. The excitation spectrum (λ_em_ = 600 nm) showed the overlapped absorption bands of the PDI and of the Ru complex. Transient absorption spectroscopy with an excitation wavelength of 525 nm showed a bleaching at 530 nm, and a broad band with a maximum at 700 nm, associated with the charge separation reached through the transfer from the Ru(II) cation to the PDI, leading to the Ru(III)-PDI radical anion, being a charge-separated state whose decay led to the triplet state of the PDI with a transient absorption at 515 nm. While working with an excitation wavelength of 450 nm, the bleaching of both the PDI and the Ru complex plus the absorption band at 696 nm (related to the PDI radical anion) were observed. Additionally, in this case, the triplet state of the PDI was reached, independently on which group was excited, and the phosphorescence lifetime in the absence of oxygen was 1.8 μs. The triplet state was efficiently quenched by molecular oxygen, which was turned into its singlet state. The measured quantum yield of ^1^O_2_ was 57% and its phosphorescence lifetime was 70 μs. Intramolecular electron transfer processes were studied in the presence on triethylamine (TEA) as a chemical sacrificial electron donor and after degassing to remove oxygen. In the dark, TEA was added to a solution of **17** in acetonitrile in a 1:1 or 1:2 ratio, and the changes in the absorption spectrum showed a reduction in the intensity of the PDI bands, together with the concomitant growth in the absorptions in the near IR (704, 796 and 953 nm); hence, the complex could oxidize the TEA and form a PDI radical anion, which was stable in the absence of oxygen, with the orange solution becoming green. The irradiation of the green solution with 420 nm light brought about the formation of the PDI dianion, stable in the absence of oxygen, and the change in color to violet. Finally, in the presence of oxygen, the initial **17**-TEA orange solution was restored, indicating its ability to act as an electron acceptor. Compound **17** showed interesting spectroscopic properties and the ability to form ^1^O_2_, and the experiments with TEA demonstrated an efficient formation of PDI radical anions that could be useful in organic synthesis.

In 2017, Campagna, Nastasi, Prato et al. [50] published the Ru–PDI complex **18** (see Figure 8, data in Table 3), the perylene subunit of which was characterized by a bay-unsubstituted perylene core and two phenanthroline ligands in imidic positions; furthermore, four bipyridines were present to chelate two Ru(II) cations.

As with compound **17**, **18** had three absorption bands between 450 and 550 nm, associated with the PDI absorption and with a contribution of MLCT transitions between 350 and 470 nm. From electrochemical measurements it emerged that **18** had two reversible reductions at −0.37 and −0.61 V vs. SCE and an oxidation at +1.31 V associated with the metal center. No oxidation of the PDI was observed, because the metal cation moved it to more positive potentials. Differently from the free ligand, being a good emitter characterized by two emissions at 535 and 575 nm, a quantum yield of 91% and a lifetime of 4.3 ns, compound **18** was not emissive. The luminescence quenching arose from the intersystem crossing promoted by the heavy metal cations, the photoinduced reductive electron transfer leading to the formation of a charge-separated state, and the MLCT transitions. Transient absorption experiments showed the features of π-π* transitions of the PDI at the short timescale (100 fs), that were the bleaching of the absorption of the PDI and the transient absorption at λ > 630 nm, suggesting an efficient energy transfer from the ^1^MLCT state to the ^1^π-π* level. The formation of the PDI radical anion deriving from the photoinduced reductive electron transfer from the Ru complex to the PDI was confirmed by a band between 620 and 720 nm, followed by a charge recombination yielding the ^3^π-π* PDI, which decayed after a final intersystem crossing. Compound **18** has interesting spectroscopic and redox properties, and the design of novel PDI–phen–Ru(II) complexes could lead to a family of compounds with even better properties.

The previously cited work of Chao, Gasser et al. [40] also described the application of complex **6b** (structure in Figure 4, data in Table 1) as a sensitizer for PDT.

Even in this case, the first step was the assessment of the stability of **6b** through ^1^H-NMR spectroscopy after 4 h of incubation, which did not show any sign of degradation. Furthermore, the photochemical behaviour was explored by irradiating a solution of **6b** in PBS at 420 nm for 15 min, which confirmed its photostability. Then, the ability to generate ^1^O_2_ was determined in acetonitrile through an indirect method, and **6b** showed a lower quantum yield (29%) than that of **6a** (85%), thus, indicating a lower capability to generate singlet oxygen. Additionally, in this case, the poor solubility of **6b** in PBS provoked a reduction in the quantum yield (4%). The dark and the phototoxicity indicated IC_50_ values higher than 20 μM for the former and ranging from 9.4 ± 1.6 and 10.4 ± 0.9 μM for the latter, resulting in phototoxic indexes of about 2. Confocal microscopy was unable to determine the cellular uptake of **6b**, but through ICP-MS analysis a concentration of 1.54 nmol per mg of protein was detected, mostly in the nucleus (1.26 nmol per mg of protein). Compound **6b** showed a moderate phototoxicity and its dark toxicity was associated with the higher cellular uptake and with its accumulation mostly in the nucleus.

In 2018, Carlos et al. [54] tested the previously described compound **17** (structure in Figure 8, data in Table 3) as a sensitizer for PDT. Such a compound was chosen because of its intense absorption and emission in the visible region and in the near IR, a long emission lifetime deriving from the triplet state of the PDI (1.8 μs), and a high singlet oxygen quantum yield (70%).

The absorption and emission spectra in DMSO were characterized by the contributions of both the PDI and the Ru(II) complex, with a weak absorption band at around 700 nm related to the formation of the PDI radical anion; the emission spectra also consisted of the contributions of the two chromophores. Complex **17** showed a photoreactivity associated with the formation of the PDI radical anion, and when the light was turned off, the initial compound was restored. Different spectroscopic properties were observed when **17** was characterized in RPMI-1640 medium, and a marked aggregation and an intensity inversion of the 0-0 and 0-1 bands were observed. Furthermore, the emission spectrum was characterized by a remarkable aggregation, by the complete quenching of the PDI emission, and by the prevalence of the MLCT transition of the Ru complex. As the DMSO solution, complex **17** in in RPMI-1640 also showed the formation of the PDI radical anion and its reverse reaction after turning off the light. Comparable results were obtained for a buffer solution (pH 7.4) of **17**, and more unstructured spectra in the case of starch films containing **17**, with a 50 nm red shift of the emission band associated with strong molecular interactions. Singlet oxygen was quantified by employing a solution of 2,2,6,6-tetramethyl-4-piperidinol (TMP-OH), being oxidized to the corresponding N-oxide (4-hydroxy-2,2,6,6-tetramethylpiperidine-N-oxyl, TEMPOL) by ^1^O_2_, in buffer solution (pH 7.4) and working with 420 and 518 nm excitation wavelengths; as a result, in both cases the formation of ^1^O_2_ took place. After 20 min of irradiation, the concentration of singlet oxygen ranged from 0.03 to 0.27 μM with λ_ex_ = 518 nm and from 0.02 to 0.31 μM with λ_ex_ = 420 nm, passing from a 10 to a 150 μM solution of **17**. Complex **17** was incubated for 24 h with B16F10-Nex2 cells to evaluate the dark toxicity; the IC_50_ had a value of 28 μM and the dark toxicity was negligible. On the other hand, a phototoxic effect was observed when irradiating for 5 min with 420 nm light (3.52 J cm^−2^) and observing the effects after 24 h. The IC_50_ dropped to 0.5 ± 0.16 μM and appreciable changes in the cell morphology could be seen (cells became smaller and rounded); furthermore, the irradiation for 30 min with 518 nm light (0.41 J cm^−2^) also provoked a reduction in the IC_50_ to 1.2 μM. Complex **17** shows not only interesting spectroscopic properties, but even a good ability to generate singlet oxygen together with a very low dark toxicity, which are important aspects when considering a compound for PDT.

In 2018, Maia, Selke et al. [51] published the new ruthenium (II) perylene complex **19** (see Figure 8, data in Table 3), having the same structure of complex **17** except for the functionalization of the perylene core; in fact, only one imidic position was present and functionalized with the phen, leaving the other side as an anhydride. This compound was characterized and tested as a sensitizer for the photodynamic inactivation of *Candida Albicans* fungi.

The electrochemical characterization of **19** showed two reductions at −0.15 V vs. Ag/AgCl, and −0.52 V, assigned to the PDI radical anion and dianion, and another two at −1.07 and −1.15 V assigned to the Ru(II) center. A high irreversible oxidative potential of +1.17 V was associated with the Ru(II)/Ru(III) oxidation, much higher than that of the simpler precursor *cis*-RuCl_2_(phen)_2_ (+0.43/+0.35 V) because of the electron-withdrawing perylene ligand. Similar to the other PDI–Ru assemblies, the absorption spectrum of complex **19** was that of the free PDI ligand plus that of the [Ru(phen)_3_]^2+^ analogue, with two distinct bands around 490 and 530 nm (π-π* of PDI) and a broad absorption centered at about 450 nm (MLCT of Ru^2+^). The emission spectrum with λ_ex_ = 413 nm (in the region of the MLCT transition) showed a broad band between 450 and 700 nm, while when exciting at 460 and 495 nm (in the region of the PDI transition) two peaks at 550 and 584 nm were observed. The singlet oxygen formation was measured by direct analysis, by irradiating the sample with 532 nm light and by measuring the ^1^O_2_ quantum yield, which was around 23%. The cytotoxicity of **19** was assessed in mouse macrophages, finding a high value above 25 μM; hence, the selected concentration for the antimicrobial photodynamic therapy tests was 12.5 μM. A small reduction in *C. albicans* was observed when incubated with **19** without irradiation, and a more pronounced reduction when irradiated with 532 nm light.

**Figure 9:**
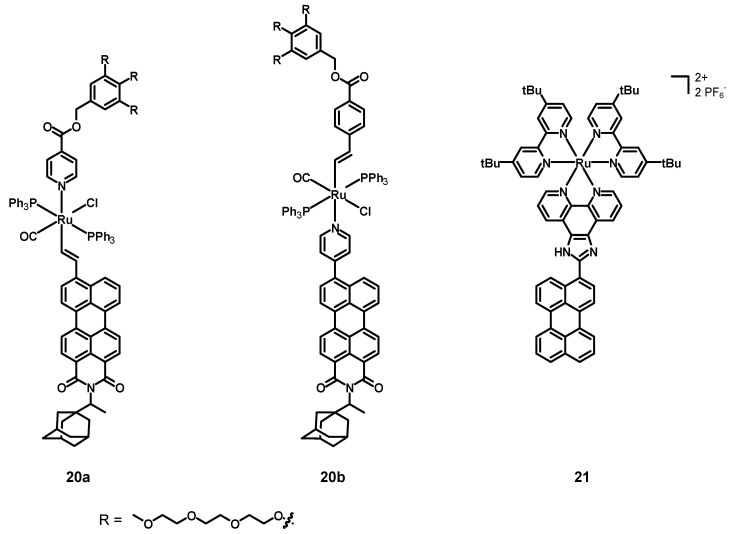
Structures of complex **20a**–**b** and **21**.

In 2019, Ely, Torroba et al. [52] synthesized, characterized, and tested the two alkenyl perylene-Ru(II) complexes **20a**–**b** (structures in Figure 9, data in Table 3) as detectors for toxic vapor and gases. These compounds were constituted by a Ru center coordinated to a chloride anion, a carbon monoxide molecule, and two triphenylphosphines. The remaining ligands were a pyridine and an alkene connected with a fluorophore and some groups aimed at increasing the solubility. The former compound was a perylene monoimide with a bulky adamantyl group in the imidic position (to increase the solubility), and the latter contained three triethylenglycol monomethyl ether chains to improve the solubility in water.

The UV–Vis spectra of **20a**–**b** in DCM were characterized by intense absorption maxima at 575 and 500 nm. The emissions were characterized by broad bands between 500 and 700 nm, with quantum yields of 38 and 29%, and lifetimes of 4.74 and 4.59 nm, respectively. Moreover, a strong bathochromic effect was observed when increasing the solvent polarity. The complexation also led to compounds with a slight solubility in water–organic mixtures and, therefore, acetone was chosen as the optimal solvent for the detection of analytes. First, the two complexes were screened in the presence of several metal cations, with slight modifications of the luminescence of **20a** in the presence of Cu^2+^ and of **19b** with Au^3+^. Anions were also screened, and only small changes were observed, except when **19a** was treated with CN^−^, because of the formation of a complex with the Ru^2+^ center. Appreciable spectroscopic changes were displayed by **20a** and **20b** in the presence of carbon monoxide and *tert*-butyl isonitrile (*t*BuNC). In particular, marked colour changes characterized compound **20a** in the presence of different analytes, with a more pronounced increase in the fluorescent emission being observed in the case of **20b**. The limit of detection (LOD) in solution for CN^−^ and *tert*-butyl isonitrile (*t*BuNC), determined by titrations, were both 0.29 μM for **19a**, while they were 0.41 and 0.12 μM for **20b**. Compounds **20a**–**b** were also immobilized on a SiO_2_ support to prepare naked eye optoelectronic devices by immersing TLC plates in a solution of the complexes at 60 °C overnight. After exposing the plates to different vapors and gases (BrCN, *t*BuNC, CO, HCl, TEA), remarkable colour and emission variations appeared. However, different colour and luminescence variations could be observed in the presence of different analytes. Cyanogen bromide, for example, produced a weak luminescence and reached a maximum after 2–3 h, whereas *tert*-butyl isonitrile provoked a significant increase in the luminescence after only 30 min. Thus, two perylene monoamide–Ru complexes with a water-solubilizing unit were successfully synthesized and characterized; the appreciable changes in the colour and in the luminescence of **20a**–**b**, both in solution and when immobilized on a silica substrate, demonstrate the possibility to employ these systems for the detection of several pollutants in the vapor and gas phases.

In 2022, Rau, Tschierlei et al. [53] synthesized, characterized, and tested the perylene–Ru(II) complex **21** (see Figure 9, data in Table 3). This compound was analogous to the abovementioned compound **15**, but this time the 2,2′-bpy ligands had additional *t*Bu substituents in positions 4 and 4′ (tbbpy).

The electrochemical properties of **21** in DMF consisted of a reversible oxidation of Ru(II) to Ru(III) at 0.82 V vs. Ag/AgCl, together with two irreversible ones at +0.55 and +1.10 V (perylene). Six reductions at −1.88, −2.10, and −2.47 V (phen and bpy ligands), −1.76 V (imidazole), and 2.70 and 2.93 V (irreversible, perylene, respectively) were also detected. The UV–Vis spectrum was characterized by an intense and broad absorption peak at 467 nm (ε = 50.3 × 10^3^ M^−1^ cm^−1^) corresponding to the overlap between the perylene π-π* and the MLCT absorptions, and red-shifted of 10 nm with respect to the free perylene ligand. The other two strong absorption bands at 257 and 288 nm (due to the tbbpy ligands) were also present. The emission spectrum (λ_ex_ = 468 nm) was constituted by a single band centered at 617 nm with a low quantum yield of 1%. Moreover, the effects of the imidazole protonation and deprotonation were studied in the presence of trifluoroacetic acid (TFA) and tetrabutylammonium hydroxide (TBAOH). The protonation induced only small variations in the absorption but provoked a red-shift of 20 nm of the emission maximum. Larger effects on both the absorption and emission bands were caused by the deprotonation; the first was red-shifted of 22 nm and accompanied by the formation of a shoulder between 550 and 750 nm, while the second one was quantitatively quenched. Two emission lifetimes of 380 and 1500 ns were determined, the first being associated with ^3^MLCT transitions of the Ru(II) center, the second one with the ^3^MLCT and ^3^IL of the perylene core. Transient absorption spectroscopy suggested that the long-lived excited states were triplets, populated after charge separation and recombination processes involving a perylene radical anion or cation. The ability of compound **21** to generate singlet oxygen was assessed by measuring the ^1^O_2_ quantum yield; this complex allowed us to measure an excellent singlet oxygen quantum yield of 80 ± 6% and, therefore, it is not only interesting from the photophysical viewpoint, but also represent a promising candidate for applications in PDT.

## 4. Conclusions

This review shows and summarizes the development, characterization, and applications of iridium and ruthenium complexes bearing various perylene ligands.

In some cases, the metal center and the perylene core can be seen as two different subunits connected through covalent bonds, while in other cases the perylene core and the metal center are directly linked to each other. Independent of the shape of these complexes, the absorption spectra and the electrochemical properties can be summarized as those of the perylene subunit plus those of the metal moiety. In fact, in many cases, the absorption spectra are exactly the sum of those of the free perylene ligand (characterized by strong and sharp absorption bands in the visible region) and those of the Ir or Ru moiety, consisting of strong π-π* transitions in the UV region plus broad MLCT bands originating thanks to the heavy metal center. A similar conclusion can be pointed out for the electrochemical properties which are remarkably different for the free perylene ligand and for the complex with a perylene directly connected to the metal center.

Whereas the absorption and electrochemical properties of the perylene moiety are not strongly affected by the presence of a metal center, the emissions are usually completely changed. Differently from fully organic PDIs, characterized by a strong fluorescence in the visible region, lifetimes of few nanoseconds, and luminescence quantum yields approaching 100%, perylene Ir and Ru complexes show virtually no emissions. In few cases, weak phosphorescent emissions with lifetimes in the range of microseconds were reported. This was due to a strong interaction between the perylene core and the heavy metal center taking place after the excitation, leading to charge separation and charge transfer states, and finally to an excited PDI in its triplet state, which deactivated mostly through non-radiative mechanisms promoted by the metal.

Another important point is represented by the strongly red-shifted absorption spectra when perylenes are used as ligands, especially for applications in biological environments, because of the stronger penetrating power and the reduced detrimental effects associated with the use of longer wavelengths in the excitation radiations.

However, despite the red-shifted absorption spectra, the poor emission properties after the coordination to the metal centre with respect to the highly emissive perylene ligands alone clearly represent an important limitation in applications, such as bioimaging, in which a strong emission intensity is necessary for a proper visualization. Nevertheless, the advantage deriving from the presence of the heavy metal centre plus the red-shifted absorption spectra is represented by the ability to generate large amounts of singlet oxygen, which is the main active species for photodynamic therapy.

Furthermore, another limitation is the electron-poor character of the perylene core when perylene–Ru dyes are employed as sensitizers for DSSCs; the electron-poor character of the PDI connected to the ancillary ligand and the absence of conjugation between the two strongly limit the photovoltaic performances of the device. For this kind of application, the design of ancillary ligands functionalized with electron-richer perylene subunits instead of PDIs is highly recommended.

The abovementioned studies unveil interesting iridium and ruthenium complexes bearing a perylene moiety, which are appealing for their possible applications in LECs, PDT, DSSCs, and in the detection of pollutants. Their further development could likely lead to highly performing dyes and promising candidates for these applications.

## Figures and Tables

**Table 2:** Photophysical and redox properties of complexes **9**, **10**, **11**, **12**, **13a**–**d**, **14a**–**e**, **15a**–**c**, and **16**.

Complex	λ_abs_ (nm) (ε, M^−1^ cm^−1^)	λ_em_ (nm) (Φ (%); τ)	E_red_ (V)	E_ox_ (V)	Ref.
**9**		620 (0.1; -) ^1^	−0.62 (rev.) ^2,3^ −0.77 (rev.) ^2,3^	+1.6 (irr.) ^2,3^ +0.81 (rev.) ^2,4^ +1.36 (rev.) ^2,4^	[41]
**10**	647 ^5^				[42]
**11**	296 ^6^ 652 ^6^	680 (0.1 ^3^, 0.011 ^4^, -)	−0.6 (rev.) ^3,7^	+0.74 (rev.) ^4,7^ +1.42 (rev) ^4,7^	[43]
**12**	447 (16,800) ^8^ 540 (29,900) ^8^ 580 (45,000) ^8^			+0.87 ^4,8,9^ +0.92 ^8,10,11^	[44]
**13a**	438 ^4,12^ 650 ^3,12^	750 (0.33; 57 μs)	−0.67 ^11^ −0.87 ^11^ −0.99 ^11^	+0.90 ^11^ +1.38 ^11^	[45]
**13b**	438 ^4,12^ 650 ^3,12^	750 (0.39; 35 μs)	−0.67 ^11^ −0.87 ^11^ −1.00 ^11^	+0.94 ^11^ +1.40 ^11^	
**13c**–**d**	438 ^4,12^ 545 ^4,12^	580 (2.0; 62 μs)	−0.55 ^11^ −0.80 ^11^ −0.97 ^11^	+1.42 ^11^ +1–70 ^11^	
**14a**	441 (41,000) ^8^ 500 (54,000) ^8^ 533 (71,000) ^8^	569 ^12^			[46]
**14b**	461 (41,000) ^8^ 492 (51,000) ^8^ 528 (62,000) ^8^	567 ^12^			
**14c**	431 (37,000) ^8^ 457 (44,000) ^8^ 520 (26,000) ^8^	583, 637 ^12^			
**14d**	453 (44,000) ^8^	469, 495 ^12^			
**14e**	488 (60,000) ^8^	538 ^12^			
**15a**	288 (1905) ^8^ 313 (177,827) ^8^ 497 (75,858) ^8^ 570 (52,481) ^8^				[47]
**15b**	288 (194,984) ^8^ 313 (199,526) ^8^ 497 (79,433) ^8^ 570 (41,687) ^8^				
**15c**	288 (199,526) ^8^ 313 (208,930) ^8^ 497 (83,176) ^8^ 570 (27,542) ^8^				
**16**	344 ^8^ 438 (692,000) ^8^	610 (0.32; 28.2 μs)	−1.22 (irr.) −1.40	+1.05 (rev.)	[48]

^1^ Measured in DCM. ^2^ Using SCE as a reference electrode. ^3^ Process associated with the perylene subunit. ^4^ Process associated with the metal center. ^5^ Measured in ethanol (EtOH). ^6^ Measured in chloroform. ^7^ Using a silver wire as a reference electrode. ^8^ Measured in *N,N*-dimethylformamide (DMF). ^9^ Using Ag/AgCl as reference electrode. ^10^ Dye adsorbed on a TiO_2_ film. ^11^ Using Ag/AgCl as reference electrode and converted to a normal hydrogen electrode (NHE, Ag/AgCl = 0.22 vs. NHE). ^12^ Measured in ACN.

**Table 3:** Photophysical and redox properties of complexes **17**, **18**, **19**, **20a**–**b**, and **21**.

Complex	λ_abs_ (nm) (ε, M^−1^ cm^−1^)	λ_em_ (nm) (Φ (%); τ)	E_red_ (V)	E_ox_ (V)	Ref.
**17**	450 ^1^	525, 600	−0.27 ^2,3^ −0.55 ^2,3^ −1.20 ^2,4^ −1.45 ^2,4^	+1.47 ^2,5^	[49]
**18**	524 (68,000) ^6^ 490 (48,000) ^6^ 460 (31,000) ^6^		−0.37 (rev.) ^3,7^ −0.61 (rev.) ^3,7^	+1.31 ^5,7^	[50]
**19**	493 ^8^ 533 ^8^	550, 584, 635 ^9^	−0.15 ^2,3^ −0.52 ^2,3^ −1.07 ^2,5^ −1.15 ^2,5^	+1.17 ^2,5^	[51]
**20a**	575 (50,119) ^10^	550 (38; 4.74 ns)			[52]
**20b**	500 (50,119) ^10^	550 (29; 4.57 nm)			
**21**	257 (75,800) ^11^ 286 (89,600) ^11^ 467 (50,300) ^11^	617 (1; 380 ns, 1500 ns)	−1.76 ^2,12,13^ −1.88 ^2,12,14^ −2.10 ^2,12,14^ −2.47 ^2,12,14^ −2.70 ^2,12^ −2.93 ^2,12^	+0.55 (irr.) ^2,3,12^ +0.82 (rev.) ^2,5,12^ +1.10 (irr.) ^2,3,12^	[53]

^1^ Measured in ACN. ^2^ Using Ag/AgCl as a reference electrode. ^3^ Process associated with the perylene subunit. ^4^ Process associated with the phen ligands. ^5^ Process associated with the metal center. ^6^ Measured in DMF. ^7^ Using SCE as a reference electrode. ^8^ Measured in DMSO. ^9^ Excitation wavelength (λ_ex_) of 460 or 495 nm. ^10^ Measured in DCM. ^11^ Measured in ACN. ^12^ Using Fc/Fc^+^ as an internal standard. ^13^ Process associated with the imidazole. ^14^ Process associated with the phen and the bpy ligands.

## Data Availability

Not applicable.
